# Immunization with Tp0954, an adhesin of *Treponema pallidum*, provides protective efficacy in the rabbit model of experimental syphilis

**DOI:** 10.3389/fimmu.2023.1130593

**Published:** 2023-03-09

**Authors:** Yuxing He, Dejun Chen, Yue Fu, Xinzhuo Huo, Feijun Zhao, Ling Yao, Xiuping Zhou, Pengfei Qi, Haoquan Yin, Longgu Cao, Hui Ling, Tiebing Zeng

**Affiliations:** ^1^ Institution of Pathogenic Biology and Hunan Provincial Key Laboratory for Special Pathogens Prevention and Control, Hengyang Medical School, University of South China, Hengyang, China; ^2^ Hunan Province Cooperative Innovation Center for Molecular Target New Drug Study, University of South China, Hengyang, China; ^3^ Department of Laboratory Medicine, Changsha Health Vocational College, Changsha, China; ^4^ Department of Clinical Medical undergraduates, Hengyang Medical School, University of South China, Hengyang, China; ^5^ College of Medical Imaging and Inspection, Xiangnan University, Chenzhou, China; ^6^ Hunan Province Key Laboratory of Tumor Cellular and Molecular Pathology, Cancer Research Institute, Hengyang Medical School, University of South China, Hengyang, China

**Keywords:** *Treponema pallidum*, Tp0954, placental adhesin, vaccine, immunoprotection, dissemination

## Abstract

Syphilis, a chronic multisystemic disease caused by spirochete *Treponema pallidum* subspecies *pallidum* infection, continues to be a serious global health problem and congenital syphilis remains a major cause of adverse outcomes in pregnancy in developing countries. The development of an effective vaccine is the most cost-effective way to eliminate syphilis, but so far has been elusive. Here, we evaluated the immunogenicity and protective efficacy of Tp0954, a *T. pallidum* placental adhesin, as a potential vaccine candidate in a New Zealand White rabbit model of experimental syphilis. Animals immunized with recombinant Tp0954 (rTp0954) produced high titers of Tp0954-specific serum IgG, high levels of IFN-γ from splenocytes and specific splenocyte proliferation response when compared to control animals immunized with PBS and Freund’s adjuvant (FA). Furthermore, rTp0954 immunization significantly delayed the development of cutaneous lesions, promoted inflammatory cellular infiltration at the primary lesion sites, as well as inhibited *T. pallidum* dissemination to distal tissues or organs when compared with that of the control animals. In addition, the naïve rabbits receiving popliteal lymph nodes from Tp0954-immunized, *T. pallidum*-challenged animals were not infected by *T. pallidum*, confirming sterile immunity. These findings suggest that Tp0954 is a potential vaccine candidate against syphilis.

## Introduction

Syphilis, caused by infection with spirochete *Treponema pallidum* subspecies *pallidum* (*T. pallidum*), is a chronic multisystemic sexually transmitted disease (STD). It is usually transmitted by sexual contact or mother-to-child vertical transmission during pregnancy, and sometimes *via* blood transfusion (1). Despite comprehensive efforts to control syphilis, it remains a serious global public health issue, with the most recent estimated burden of 18 million cases in the world Each year, 5.6 million people between 15 and 49 are diagnosed with this disease (2). In recent years, syphilis rates have been sharply rising among men who have sex with men (MSM) in high- and middle-income countries such as United States, China, Canada, Germany, UK and Australia (3–8). Congenital syphilis may lead to stillbirth and neonatal death (9). Furthermore, the occurrence of congenital syphilis is increasing. The number of pregnant women infected with syphilis worldwide is estimated to be 1.36 million every year, with an estimated 1.36 million pregnant women who were infected with syphilis worldwide annually, resulting in more than 0.5 million cases of adverse outcomes (10). The dramatic increase in cases of congenital syphilis is particularly worrying as it is the number one cause of fetal loss in developing countries and the second leading cause of preventable stillbirths worldwide (11). In addition, active syphilis contributes to acquiring and transmitting HIV (12). Although *T. pallidum* remains sensitive to penicillin, resistance to macrolides (e.g. erythromycin, azithromycin) which are often used as second-line alternatives to penicillins is widespread (13). The devastating consequences of congenital syphilis, the resistance of *T. pallidum* to second-line antibiotics, and the increasing prevalence of syphilis worldwide highlight the essential need to develop a syphilis vaccine as an effective complement to current public health screening and treatment programs to eradicate syphilis globally (14, 15).

Decades of research into syphilis vaccines have yielded some results. However, to date, only immunization with whole cells of γ-irradiated *T. pallidum* provided complete protection against homologous *T. pallidum* challenge in the rabbit model (16). Although such an immunization regimen is not suitable for humans, it provided feasibility for the development of a syphilis vaccine and, simultaneously, emphasized the important role of *T. pallidum* surface proteins in providing immune protection. Partial protection was obtained by immunizing with recombinant *T. pallidum* proteins or their protein-coding DNA plasmids, including 4D (17), endoflagella (18, 19), Gpd (20, 21), Tp92 (BamA) (22, 23), TprK (24), TprF (25), TprI (26), Tp0751 (15), Tp0136 (27, 28) and Tp0663 (28), based on attenuated development and rapid healing of lesions. At present, more research on syphilis vaccine candidates has focused on surface-exposed outer membrane proteins due to their direct exposure to the host immune system. However, indeed, *T. pallidum* has only rare integral proteins in its outer membrane that may help the organism escape the immune responses (29, 30).

The development of syphilis vaccine requires paying attention to the highly invasive nature of *T. pallidum* that crosses the endothelial, placental and blood–brain barriers in the early stage of infection, and then disseminates to various tissues and organs through blood and lymph fluid (29). Recent studies showed that the vaccinating rabbits with the surface-exposed lipoprotein adhesins Tp0751 and Tp0136 could prevent the dissemination of *T. pallidum* (15, 28), suggesting that the *T. pallidum* adhesins may be promising vaccine candidates.

Recently, Primus et al. demonstrated that *T. pallidum* Tp0954 is a surface lipoprotein adhesin, and further found that Tp0954 heterologously expressed on *Borrelia burgdorferi* B314 strain with poor adhesion ability facilitates its attachment to epithelial, endothelial, neuronal and placental cell lines (11). The findings above imply that Tp0954 may be an important adhesin that mediates initial infection of *T. pallidum* by colonizing mucosal epithelial cells, as well as helps its dissemination and colonization of distal sites by binding to different tissue cells, especially placental colonization and transplacental transmission (11). Moreover, Tp0954 exhibited significant reactivity with sera from syphilis patients and *T. pallidum-*infected rabbits in different stages of *T. pallidum* infection (31, 32), suggesting that Tp0954 is able to induce antibody response and to be readily expressed by *T. pallidum*. Additionally, it was determined that Tp0954 is largely conserved among Nichols, SS14, Chicago, Mexico A, and Amoy strains of *T. pallidum* with 100% identity (11). All these findings imply that Tp0954 could be a syphilis vaccine candidate against infection caused by various strains of *T. pallidum*.

In this study, we evaluated the immunogenicity and immunoprotective efficacy of Tp0954 in New Zealand White (NZW) rabbits model of experimental syphilis. The results showed the immunization with rTp0954 induced strong humoral immune responses and high levels of IFN-γ secretion from splenocytes and specific splenocyte proliferation, and delayed the development of lesions and inhibited *T. pallidum* dissemination by reducing treponemal burden in distal organs compared to that of the control animals. In addition, the naïve recipient rabbits of popliteal lymph nodes (PLNs) from Tp0954-immunized, *T. pallidum*-challenged animals were not infected by *T. pallidum*, further suggesting that Tp0954 immunization prevents treponemal seeding to distal organ sites. These findings suggest that Tp0954 is a promising vaccine candidate against *T. pallidum* infection.

## Materials and methods

### *T. pallidum* propagation and isolation

The *T. pallidum* Nichols strain was propagated in healthy male NZW rabbits by intratesticular inoculation and harvested as previously described by Lukehart et al. (33). All rabbits were screened by rapid plasma reagin test (RPR) and *Treponema pallidum* particle agglutination assay (TPPA) to exclude *T. paraluiscuniculi* infection.

### Recombinant protein expression, purification and identification

For expression of rTp0954 protein in *Escherichia coli*, *tp0954* gene (GenBank accession number AAC65909.1, 1437-bp full length) coding full-length Tp0954 protein with 478 amino acid residues without the N-terminal signal peptide (19 amino acid residues) and with hexahistidine tag sequence, was PCR amplified from *T. pallidum* (Nichols strain) genomic DNA using the Sense: 5′-ATGGGATCCTGTGTTAGCACCGGTTCTAACTCTG-3′ and Antisense: 5′-CATCTCGAGTTACGGGTTGGTACGCAGGGA-3′ primers with *BamH*I and *Xho*I restriction sites underlined. The amplicon was cloned into pET30a to produce the recombinant expression plasmid pET30a/*tp0954* whose sequence was confirmed by sequencing. The *tp0954* construct was transformed into the expression strain BL21 Star *E. coli* (Invitrogen, Carlsbad, CA, USA) and rTp0954 expression was induced with 0.5 mM isopropyl-β-d-thiogalactopyranoside (IPTG) for 4 h at 25°C, followed by purification with Ni-NTA columns (Qiagen, Hilden, Germany) and treatment with Detoxi-Gel™ Endotoxin Removing Gel (Pierce Biotechnology, Inc., Rockford, IL, USA) to remove endotoxin. A bicinchoninic acid (BCA) protein assay kit (CWBIO, Beijing, China) was used to determine the protein concentrations. The target proteins were separated by SDS-PAGE and transferred to a polyvinylidene fluoride (PVDF) membrane (Merck Millipore, Darmstadt, Germany). Using 6×-His Tag Mouse Monoclonal Antibody (1:1000; Proteintech, Rosemount, IL, USA) or *T. pallidum*-infected rabbit serum (1:1000; the Institute of Pathogen Biology at the University of South China) as the primary antibody, and horseradish peroxidase (HRP)-conjugated goat anti-mouse IgG (1:10,000, Proteintech) or goat anti-rabbit IgG (1:10,000, Proteintech) as the secondary antibody, respectively, protein bands were analyzed using the eECL Western Blot Kit (CWBIO) with a G:BOX Chemi XX9 (Syngene, Cambridge, UK) digital imager.

### Immunization of the animals

A cohort of 18 RPR and TPPA-seronegative male NZW rabbits (8-13 weeks old, weighing about 3.0 kg; the Department of Experimental Zoology of the University of South China, Hengyang, China) were used for immunization. The animals were randomly selected into 3 groups: PBS-immunized control group (*N* = 5), Freund’s adjuvant (FA)-immunized control group (*N* = 5), and rTp0954-Freund’s adjuvant (rTp0954/FA)-immunized group (*N* = 8). For the rTp0954/FA-immunized group, each rabbit was sedated and immunized once every two weeks for three times with 250 μg purified rTp0954 protein (1 mg/ml) mixed with an equal volume of Freund’s adjuvant system (Sigma-Aldrich, St. Louis, MO, USA; complete Freund’s adjuvant for the first immunization and incomplete Freund’s adjuvant for the booster immunizations) at each immunization. Each immunization was delivered equally as 4 subcutaneous (0.1 ml per site) injections in the necks and 2 intramuscular (0.05 ml per site) injections in the quadricep muscles. FA-immunized and PBS controls received PBS with the FA system in a 1:1 ratio or PBS alone at corresponding time points, respectively.

### *T. pallidum* challenge procedure

Two weeks after the final immunization, 11 randomly selected rabbits (N = 3, PBS control; N = 3, FA-immunized; N = 5, rTp0954/FA-immunized) were sedated, back-shaved, and cleaned with 75% ethanol. Each rabbit was challenged intradermally with 0.1 ml of 1×10^6^ freshly isolated *T. pallidum* Nichols strain per ml in 0.9% saline at each of 8 sites (each rabbit received 0.8×10^6^ *T. pallidum*). The development of lesions, including erythema, induration and painless ulceration, was monitored and then the lesion diameters were determined every 2 days.

### Analysis of rTp0954 antibody levels

At weeks 0, 2, 4, and 6 post the first immunization and prior to intradermal challenge with *T. pallidum*, blood samples were collected from ear veins of all rabbits to obtain the serum for the detection of rTp0954-specific antibody titers by ELISA. Briefly, the 96-well plates were coated with 1 μg purified rTp0954 and blocked with 5% non-fat milk in PBST. Rabbit serum was two-fold serially diluted starting from a dilution of 1:100. HRP-conjugated goat anti-rabbit IgG (1:10,000, Proteintech) was used as a secondary antibody, and binding was detected by using TMB (Tetramethylbenzidine) peroxidase substrate. After addition of stop solution, the absorbance at 450 nm (A_450_ value) was detected with a microplate reader (BioTek Instruments, Winooski, VT, USA). Each experiment was repeated three times.

### Preparation of rabbit splenocyte suspension

Two weeks after the final immunization, 7 randomly selected rabbits (N = 2, PBS control; N = 2, FA-immunized; N = 3, rTp0954/FA-immunized) were euthanized and spleens were removed for preparation of splenic cell suspension as previously described (23). Splenocytes were resuspended in RPMI-1640 medium (Sigma Aldrich, St. Louis, MI, USA) containing 10% fetal bovine serum (FBS; Biological Industries, Beit Haemek, Israel), penicillin (100 U/ml) and streptomycin (100 μg/ml) for the following detection of lymphocyte proliferation and secretion level of IFN-γ from splenocytes.

### Detection of rabbit lymphocyte proliferation

The proliferation of lymphocytes was detected by using the Enhanced Cell Counting Kit-8 (CCK-8; Beyotime, Shanghai, China) according to the manufacturer’s instructions. Briefly, rabbit splenocyte suspension at 1×10^6^ cells/well in 200 μl was stimulated with 5 μg/ml of rTp0954. In addition, splenocyte suspension cultures with 5 μg/ml of concanavalin A (Con A, Sigma Aldrich) and PBS stimulation were used as a positive control and blank control, respectively. Following the incubation for 48 h, 20 μl of CCK-8 solution was then added to each well for 4 h prior to the end of culture. The A_450_ value was detected with a microplate reader (BioTek Instruments). Independent experiments were conducted in triplicate. A_450_ value was used to express the results, and the stimulation index (SI) was calculated according to the following formula: SI(%)=At/Ac×100% (At and Ac represents the mean A_450_ value of the experimental group and the blank control group, respectively).

### Measurement of rabbit IFN-γ secretion

IFN-γ was assessed in this study because it is an indicator of Th1 response. The rabbit splenic lymphocytes collected above were cultured in 1-cm-diameter-well culture plates (Costar, Cambridge, Mass., USA) plates with at 6×10^5^ cells in 500 μl. Each well received 5 μg/ml of rTp0954 protein and PBS as blank control, followed by incubation at 37°C for 72 h. Supernatants from cultured cells were harvested for detection of the level of IFN-γ secretion in splenocytes according to the instruction of the Rabbit IFN-γ ELISA Flex kit (Mabtech, Nacka Strand, Sweden). Independent experiments were conducted in triplicate. The A_450_ value was measured using a microplate reader (BioTek Instruments).

### Extraction of *T. pallidum* DNA and quantitative real-time PCR

DNAs were extracted from tissues of *T. pallidum*-infected rabbits, including cutaneous lesions, blood, liver and spleen, by using a TIANamp Genomic DNA Kit (Tiangen, Beijing, China) following the manufacturer’s protocol. 3 tissue specimens were taken from each animal organ to ensure good reproducibility. PCR amplification was performed by using a LightCycle 96 apparatus (Roche, Basel, Switzerland). Quantitative analysis of *T. pallidum* gDNA and rabbit gDNA was achieved by using primers targeting *T. pallidum* endoflagellar sheath protein (*flaA*) gene and rabbit collagenase-1 precursor (*MMP-1*) gene, respectively. The sequences of the primers are given as followings: *flaA* Sense: 5’-GCGGTTGCACAGTGGGAG-3’, Antisense: 5’-CAGCATGGGCGACAGGAT-3’; *MMP-1* Sense: 5’-TTGCTTCTTCACACCAGAATGCTGT-3’, Antisense: 5’-GCGTGATCAGGCACTATGTAGCAAT-3’. The primers for quantitative real-time PCR (qPCR) amplification were provided by Sangon Biotech (Shanghai, China). As directed by the manufacturer, qPCR amplifications were performed in 20-μl SuperReal PreMix Plus Kit (SYBR Green) (Tiangen, Beijing, China) reaction mixture. Standard curves were generated for *flaA* and *MMP-1* by using a 10-fold dilutions in series from 10^7^ to 10^1^ reproductions of *T. pallidum* gDNA and 2-fold dilutions in series of rabbit gDNA from 100 to 0.0488 ng/μl. The efficiency was over 99%, and the R2 value was above 0.99 in every tests, respectively. The amount of gDNA in different tissues was normalized according to the lowest gDNA concentration determined by the spectrophotometric measurements. The following conditions were used for the PCR of *flaA* and *MMP-1*: 95°C for 15 min (*flaA*) or 10 min (*MMP-1*), 40 cycles of 95°C for 10 sec, 58°C (*flaA*) or 55°C (*MMP-1*) for 20 sec, and 72°C for 20 sec. Following is an analysis of melt-curves: 95°C for 10 sec, 65°C for 60 sec, and 97°C for 1 sec. Repeat each sample three times and with a no-template control.

### Histopathology

On day 21 post-challenge, each rabbit skin lesion was removed and subdivided using a 4-mm punch. The tissues taken from cutaneous lesions were fixed, embedded and sectioned for histopathologic analysis. After conventional staining with hematoxylin and eosin (H&E), the degree of inflammatory cell infiltration in each tissue section was observed and analyzed under a microscope.

### Popliteal lymph node transfer

On day 21 post-challenge, after euthanasia, the PLNs from randomly selected 2 rTp0954-immunized rabbits and 2 PBS control rabbits were removed and disrupted using a 70-μm cell sieve in a 6-well sterile plate with 0.9% saline. Four naïve anesthetized NZW rabbits (negative RPR and TPPA) were randomly selected and the strained suspension was injected into their testis with 1 ml per testis, as described previously by Lukehart et al. (33). From the 7^th^ day, signs of orchitis of rabbits were monitored every day and seroconversion (RPR and TPPA) were assayed every 7 to 10 days. The animals that showed signs of orchitis and seroconversion (positive TPPA and 1:8 RPR titer) or that that did not develop orchitis or seroconversion until day 100 were euthanized, and the testes were harvested. *T. pallidum* in testicular exudates were purified, concentrated (33) and visualized by silver staining under light microscope.

### Statistical analysis

Statistical analysis was performed with GraphPad Prism 8.0 software. Brown–Forsythe and Welch ANOVA test were used to evaluate the significance of statistical differences between the groups. Differences in *T. pallidum* load among tissues were analyzed by Ordinary one-way ANOVA and Dunnett test (*P* < 0.05 indicated a significant difference and results are reported as Mean ± SD).

## Results

### Recombinant protein expression and antibody response

**Figure 1:**
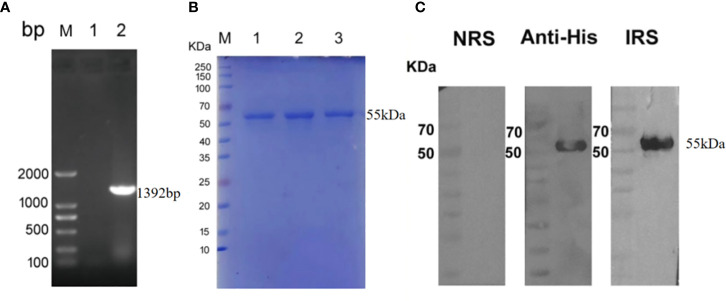
The inducible expression and identification of rTp0954 protein. **(A)** Amplification of the *Tp0954* gene from the *T. pallidum* Nichols strain genome. lane 1: negative control; lane 2: pET-30a/*tp0954*; M: DNA marker. **(B)** SDS-PAGE analysis of purified rTp0954 protein. lanes 1,2,3: purified rTp0954 protein; M: protein marker. **(C)** Western blot analysis of rTp0954 protein with normal rabbit sera (NRS), anti-His monoclonal antibody and *T. pallidum*-infected rabbit sera (IRS).

**Figure 2:**
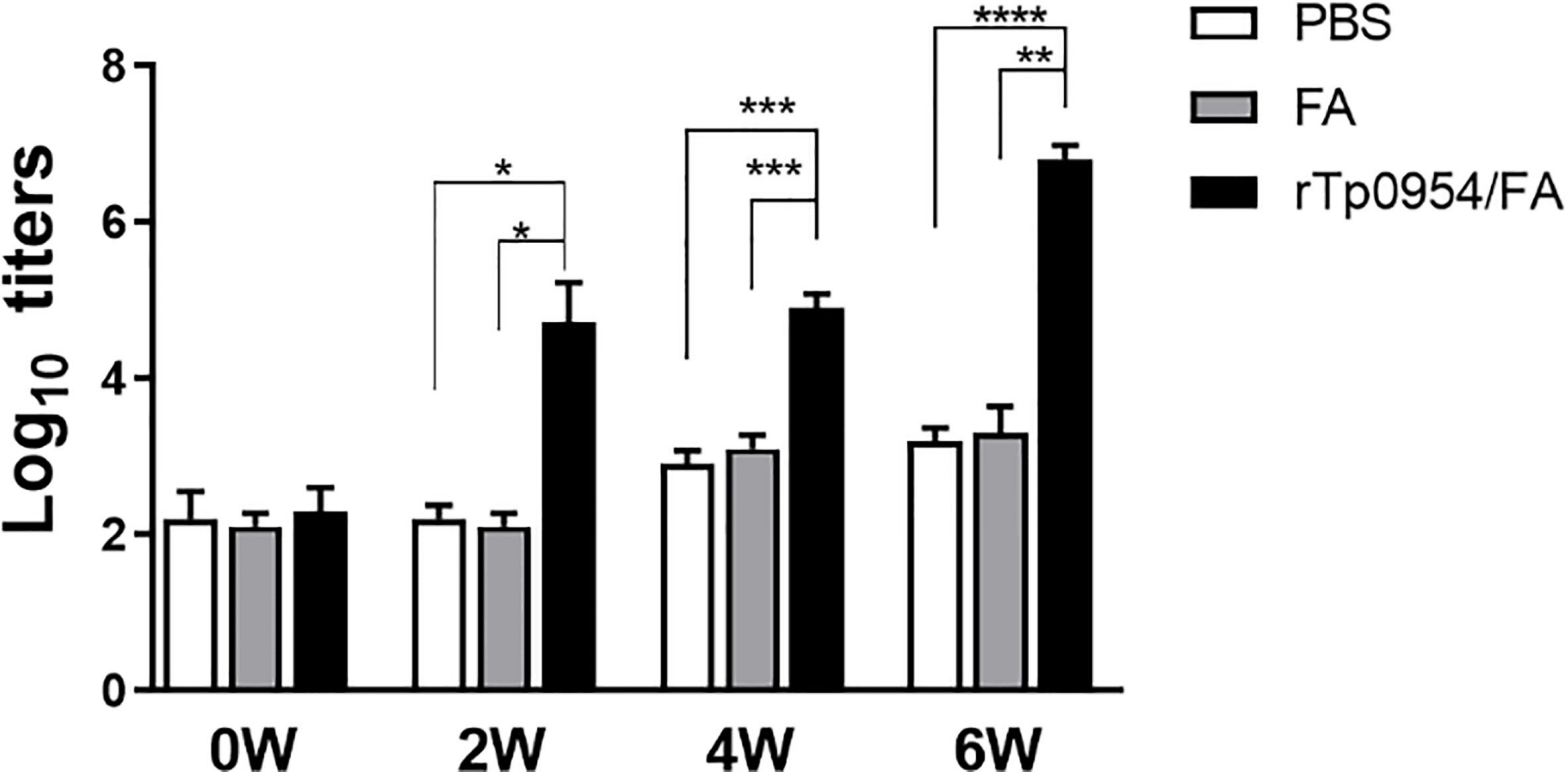
Dynamic of antibody responses to rTp0954 in immunized rabbits. Rabbits were immunized with 250 μg of rTp0954 emulsified in an equal volume of FA system, PBS with FA system in a 1:1 ratio or PBS alone as a control. Each animal was immunized once every two weeks for three times. The serum IgG titers against Tp0954 were determined by indirect ELISA at weeks 0, 2, 4, and 6 post primary immunization. The means and standard derivations for the serum IgG levels were determined from individual rTp0954-immunized (*N* = 8), FA-immunized (*N* = 5) or PBS control (*N* = 5) animals (*P < 0.05, **P < 0.01, ***P < 0.001, ****P < 0.0001).

The *Tp0954* gene was amplified from the *T. pallidum* (Nichols strain) genomic DNA (**Figure 1A**). The soluble rTp0954 protein containing the His epitope tag with approximate 55-kDa molecular mass was expressed and determined by SDS-PAGE analysis with an estimated purity of 95% (**Figure 1B**). Western blot analysis showed that target protein was only reactive with anti-His monoclonal antibody and *T. pallidum*-infected rabbit sera, not with normal rabbit sera (NRS) (**Figure 1C**). ELISA results showed that, beginning on week 2, rTp0954 induced gradually increasing titers of anti-Tp0954 IgG antibodies compared with PBS or FA-immunized control rabbits (**Figure 2**), indicating that immunization of rabbits with rTp0954 generates a specific antibody response.

### rTp0954 immunization stimulates lymphocyte proliferation

**Figure 3:**
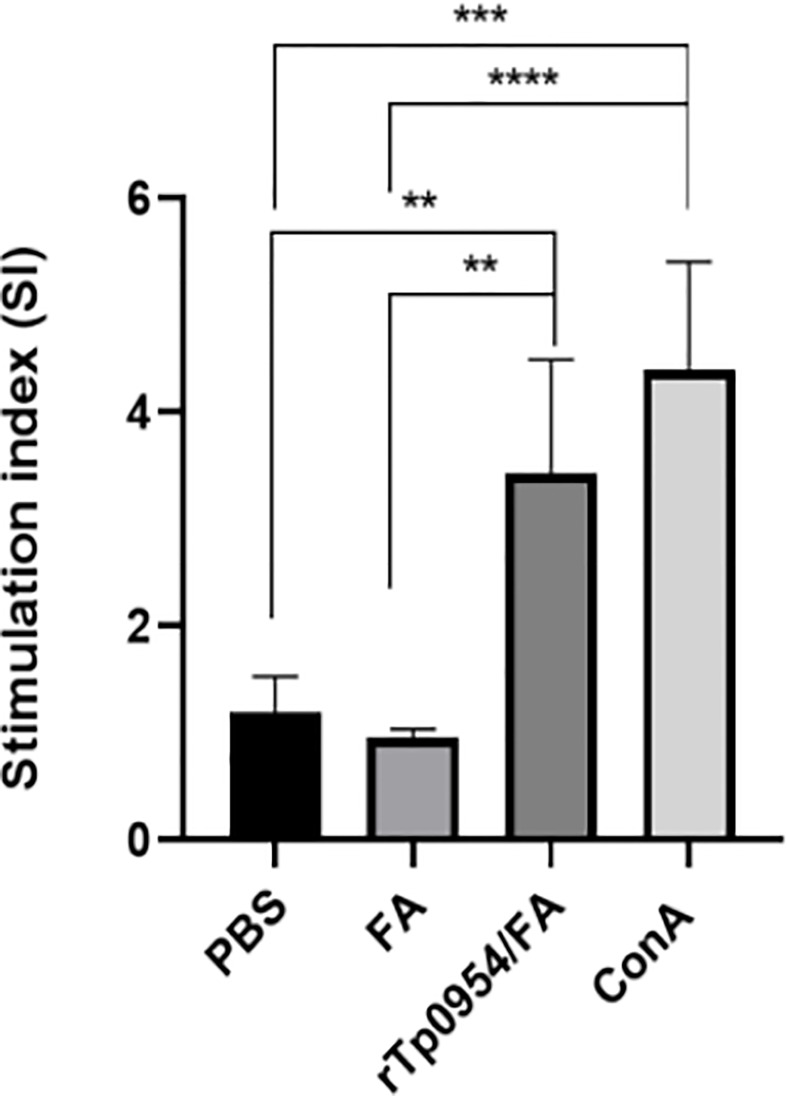
Proliferative responses of splenic lymphocytes specific to rTp0954 in different immunized rabbits. Two weeks post the final immunization, rabbit splenic lymphocytes were isolated from three groups and their proliferative responses specific to rTp0954 were measured by CCK-8 assay, with Con A and PBS stimulation as a positive control and blank control, respectively. Proliferative responses of rTp0954-specific lymphocytes were expressed as SI values. The data represent three independent experiments, expressed as the means ± SDs from individual rTp0954-immunized (*N* = 3), FA-immunized (*N* = 2) or PBS control (*N* = 2) rabbits (**P < 0.01, ***P < 0.001, ****P < 0.0001).

Two weeks after the final immunization, rabbit splenic lymphocytes were isolated from different groups and their proliferative responses specific to rTp0954 were measured by CCK-8 assay, with Con A and PBS stimulation as a positive and blank control, respectively. As shown in **Figure 3**, the positive (Con A) control showed the highest SI values, and the SI values in rTp0954-immunized groups were significantly higher than those in PBS- or FA-immunized groups (*P* < 0.01), suggesting that rTp0954 immunization induces significant proliferation of splenic lymphocytes.

### rTp0954 immunization induces IFN-γ secretion

**Figure 4:**
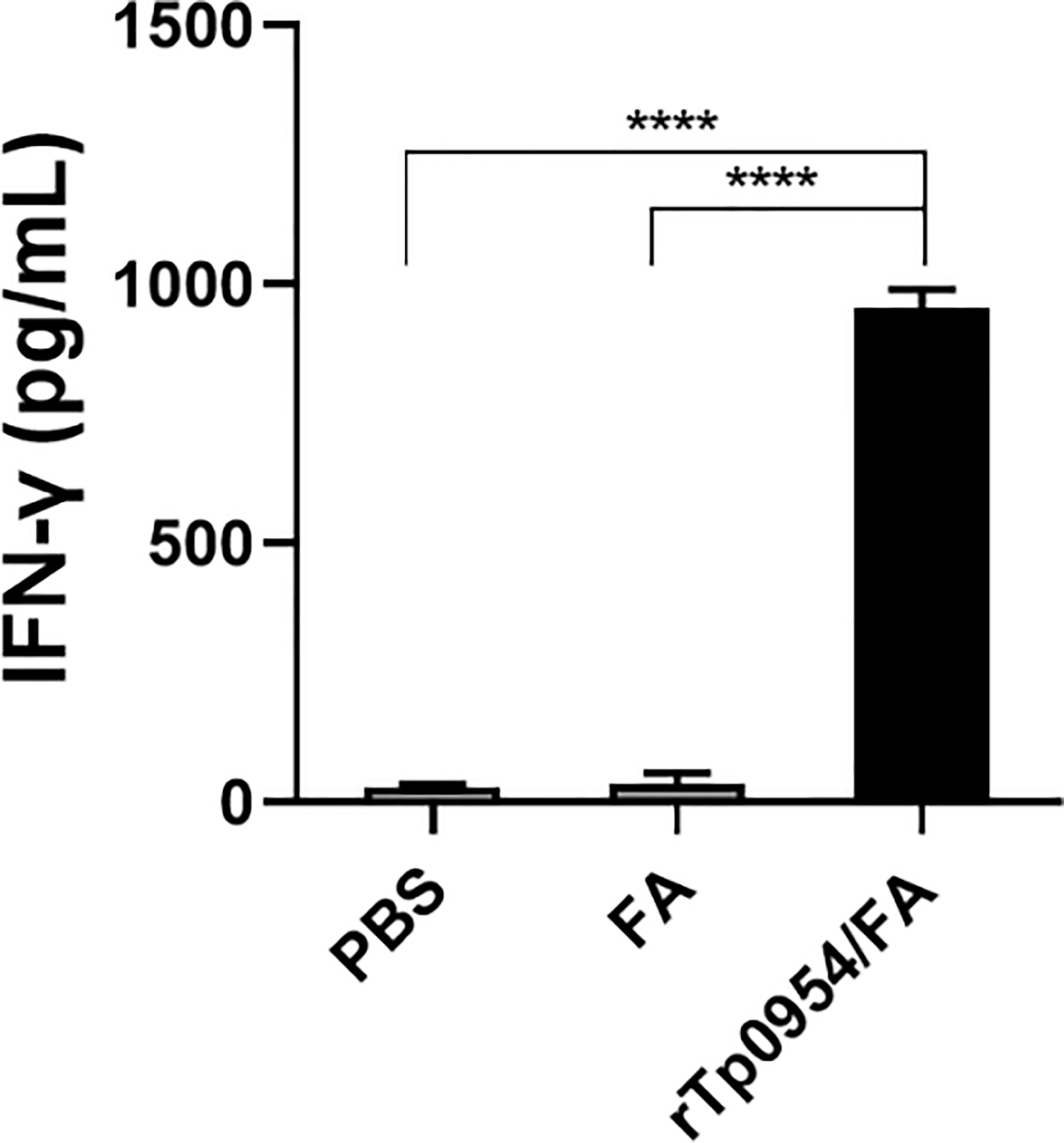
The levels of IFN-γ secreted by splenocytes stimulated with rTp0954 in different immunized rabbits. Two weeks post the final immunization, rabbit splenic lymphocytes were isolated from three groups and the levels of IFN-γ by ELISA in splenocytes stimulated with rTp0954 protein, with PBS stimulation as blank control. Results are expressed as the means ± SDs of rTp0954-immunized individual (*N* = 3), FA-immunized (*N* = 2) or PBS control (*N* = 2) animals (****P < 0.0001).

IFN-γ is a critical Th1-related cytokine, which is essential for early clearance of *T. pallidum* in lesions. The levels of IFN-γ produced by rabbit splenic cells were measured by ELISA. As shown in **Figure 4**, IFN-γ levels in rTp0954-immunized groups were significantly increased when compared with those in PBS- or FA-immunized groups (*P* < 0.0001), which closely resembles the proliferative response of splenic lymphocytes specific to rTp0954.

### rTp0954 immunization attenuates lesion development

**Figure 5:**
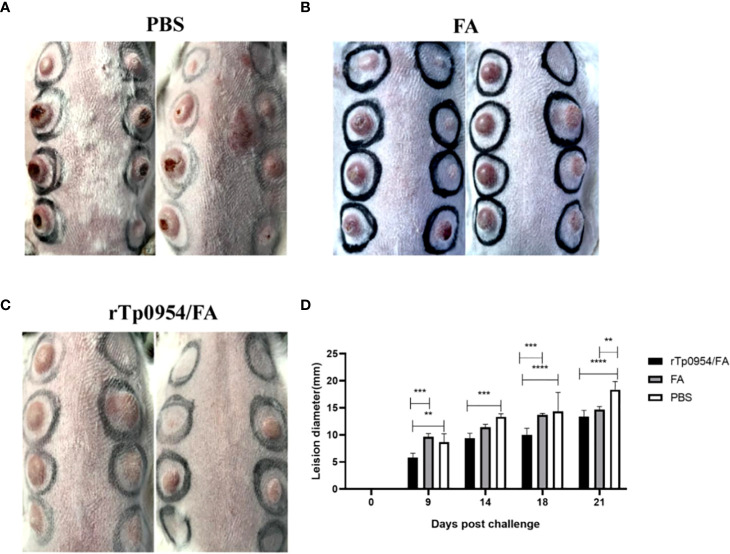
Immunization with rTp0954 protein attenuates lesion development. During the subsequent 21 days post *T. pallidum* infection, cutaneous lesions were monitored daily for diameter, induration and ulceration. The rTp0954/FA-immunized rabbits had lower incidence of ulcerative lesions and smaller lesion diameter than the FA-immunized or PBS controls on day 21 post-challenge **(A–C)**. The diameter of cutaneous lesions in the rTp0954-immunized animals was consistently smaller than that in the PBS and FA control animals over the 21-day measurement period **(D)**. The results are expressed as the means ± SDs between individual rTp0954/FA-immunized (*N* = 5), FA-immunized (*N* = 3) or PBS (*N* = 3) control animals. Two-way analysis of variance test for significance analysis (** *P* < 0.01, *** *P* < 0.001, **** *P* < 0.0001).

2 weeks post the final immunization, each animal was infected at 8 sites on the back with a total of 0.8 × 10^6^ of freshly isolated *T. pallidum*. Since the painless indurated lesion (chancre), that develops at the site of initial infection is the first clinical sign characteristic of early syphilis, monitoring the development of chancre provides a measure of the effectiveness of syphilis vaccine. During the subsequent 21 days, lesions were monitored daily for diameter, induration and ulceration. The observed results showed that rTp0954/FA-immunized animals exhibited delayed lesion development. On day 9, there were 21 and 20 lesion indurations (a total of 24) observed in FA-immunized and PBS control animals, respectively. However, by day 14, only 24 lesion indurations (total of 40) were observed in animals immunized with rTp0954/FA (**Table 1**). Moreover, on day 21 post-challenge, the incidence of ulcerative lesions in rTp0954/FA-immunized animals (12.5%) was much lower than that in PBS control (66.7%) and FA control animals (60.0%) (**Figures 5A–C**; **Table 1**). Additionally, over the 21-day measurement period post-challenge, the diameter of cutaneous lesions in the rTp0954-immunized animals was consistently smaller than that in the PBS and/or FA control animals (**Figure 5D**). During the measurement period of 9-21 days, the mean lesion size in the rTp0954-immunized animals was much smaller than that in the PBS and FA control animals(**Table 1**). All these results indicate that immunization of rabbits with rTp0954 is able to significantly delay lesion development and decrease the diameter of cutaneous lesions.

**Table 1:** Lesion data for control and immunized animals.

Groups	No. of lesions/no. of challenge sites (%)^a^	No. of ulcerative lesions/no. of lesions (%)^b^	Median lesion diameter (mm)^c^
PBS (*N* = 3)	21/24 (87.5)	14/21 (66.7)	13.67 ± 3.84
FA (*N* = 3)	20/24 (83.3)	12/20 (60.0)	12.37 ± 1.99
rTp0954/FA (*N* = 5)	24/40 (60.0)	3/24 (12.5)	9.65 ± 2.85^d^

a The denominator indicates the total number of challenge sites in each treatment group.

b The denominator indicates the total number of lesions in each treatment group.

c The mean lesion size of lesions (Mean ± SD) in each treatment group during the measurement period of 9-21 days.

d rTp0954/FA vs PBS or FA, P<0.0001.

### rTp0954 immunization inhibits *T. pallidum* dissemination

**Figure 6:**
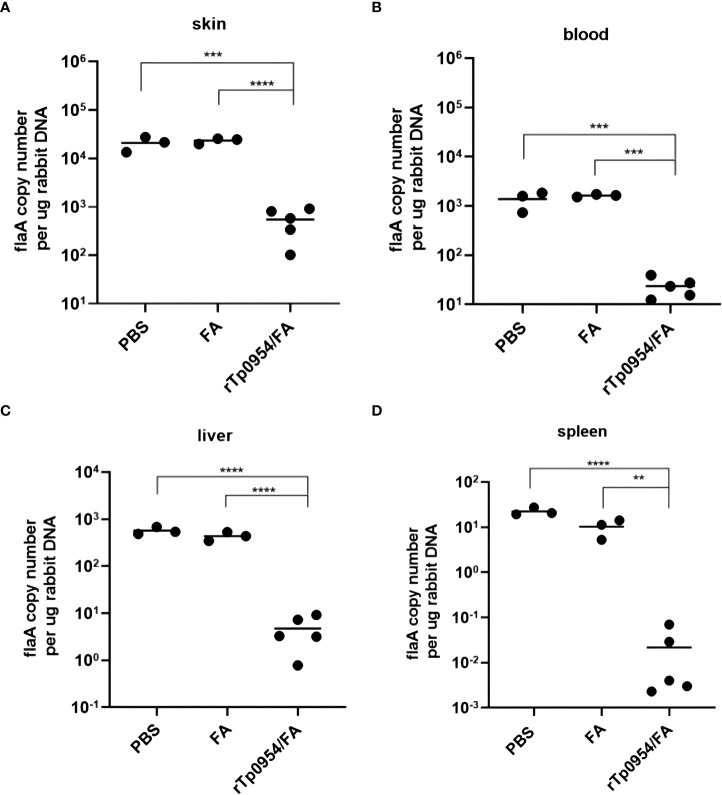
Immunization with rTp0954 protein prevents the spread of *T. pallidum*. Load of *T. pallidum* in animals immunized with rTp0954/FA (*N* = 5), FA (*N* = 3) and PBS (*N* = 3) was measured by qPCR for the *flaA* DNA concentrations in the biopsy specimens from local cutaneous lesions **(A)** and distal spread infection locations of blood **(B)**, liver **(C)** and spleen **(D)**. Results of each tissue type were normalized according to the concentration of rabbit gDNA, expressed as median ± interquartile range. Mann-Whitney U test for significance analysis (**P < 0.01, ***P < 0.001, ****P < 0.0001). Repeatability analysis was performed on three tissue samples of each rabbit organ. Each dot represents the mean *flaA* DNA concentration of three individually selective tissue samples from every organ of each rabbit. Horizontal lines represent the means of individual rTp0954-immunized (*N* = 5), FA-immunized (*N* = 3) or PBS control (*N* = 3) animals.

To evaluate the ability of rTp0954 immunization to prevent *T. pallidum* dissemination, all animals challenged with mobile *T. pallidum-*were euthanized on day 21 post-challenge. qPCR was performed to determine *T. pallidum* DNA concentration to assess the treponemal burdens in biopsy specimens obtained from cutaneous lesions at the initial infection sites as well as distal tissues or organs, including blood, spleen and liver. The results revealed that there was a noticeable decline in the *T. pallidum* DNA concentration at the initial infection sites in the rTp0954/FA-immunized animals compared to the FA and PBS controls (*P* < 0.0001) (**Figure 6A**). Owing to highly invasive ability of *T. pallidum* that spreads through the blood to various distal tissues and organs where treponemes could be detected (15, 19, 28, 34), the treponemal burdens in the blood, liver, and spleen of the animals were also assessed. As shown in **Figures 6B–D**, the treponemal burdens in the blood, livers, spleens from rTp0954-immunized rabbits were still obviously decreased than that in the FA and PBS controls (*P* < 0.01, 0.001 or 0.001), suggesting that immunization of rabbits with rTp0954 prevents treponemal dissemination to distant organs.

In order to further determine the ability of rTp0954 immunization to prevent treponemal dissemination, PLNs from 2 PBS control and 2 Tp0954/FA-immunized animals were transferred into 4 naïve recipient animals. The infection status of the rabbits receiving PLNs was determined by orchitis development, seroconversion (RPR and TPPA) and microscopic observation of silver stained-treponemes in testicular aspirates. Two PBS control naïve recipient rabbits (R-Ct1 and R-Ct2) developed overt orchitis and stronger seroconversion (1:8 RPR titer and positive TPPA) on day 40 and day 56 post transfer (**Table S1**), respectively. Silver-stained treponemes under microscopy in the concentrated testicular extracts from R-Ct1 and R-Ct2 also were observed (**Figure S1A**). Conversely, two naïve PLN recipients from the Tp0954/FA-immunized rabbits (R-Im1 and R-Im2) developed neither orchitis nor seroconversion (**Table S1**). Moreover, no treponemes were observed in testicular exudates by silver staining 100 days after transfer (**Figure S1B**). All the findings suggest that rTp0954 vaccination prevents *T. pallidum* dissemination in rabbits.

### rTp0954 immunization promotes the inflammatory infiltration

**Figure 7:**
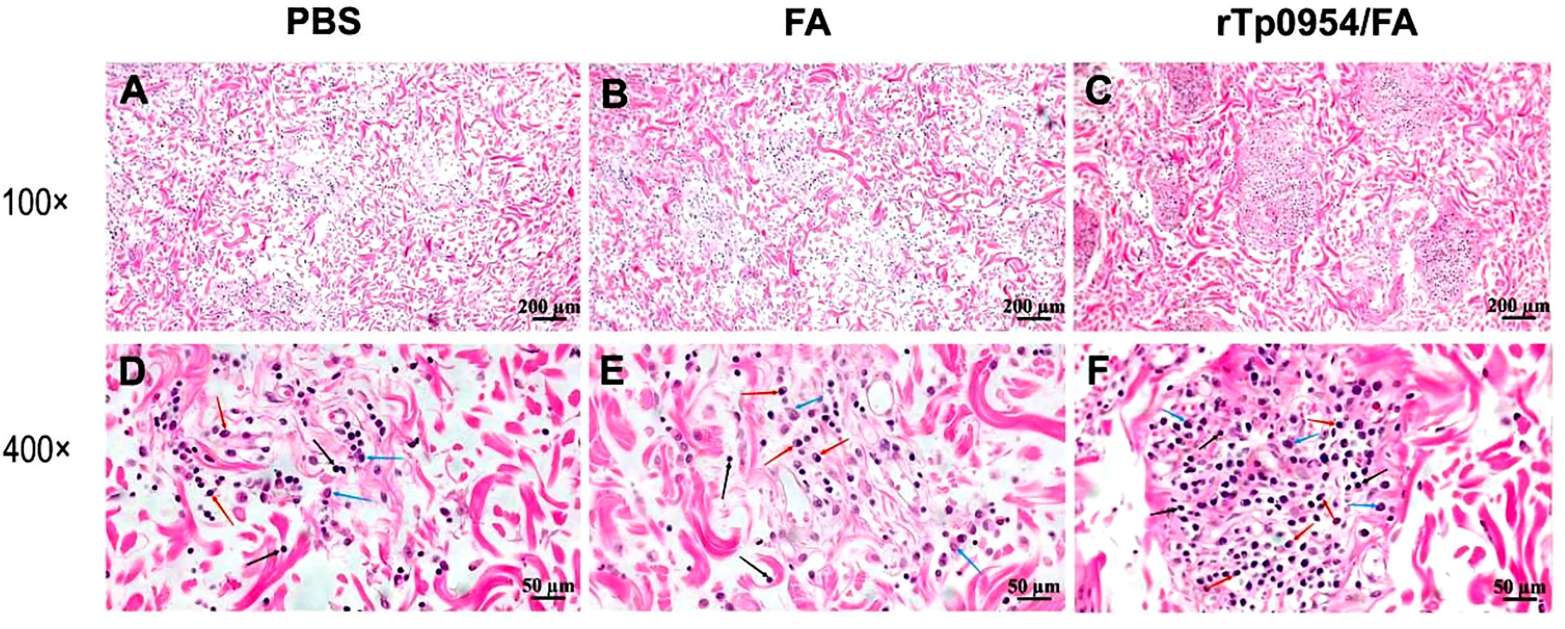
Histopathological analysis of primary cutaneous lesion sites post *T. pallidum* challenge in animals. On day 21 post *T. pallidum* challenge, cutaneous tissues from PBS- **(A, D)**, FA- **(B, E)**, or rTp0954/FA-immunized rabbits **(C, F)**, separately, were sliced and stained with H&E. PBS- and FA-immunized animals were used as controls. All lesions revealed various degrees of infiltrates of lymphoplasmacytic cells characteristic of syphilis (black arrow = lymphocyte; red arrow = plasma cell; blue arrow = macrophage).

On day 21 post-challenge, H&E stained sections from primary cutaneous lesion sites of rabbits were performed to evaluate the intensities of inflammatory infiltration and cell types. All lesions revealed various degrees of infiltrates of lymphoplasmacytic cells (mainly including lymphocytes, plasma cells, and macrophages), characteristic of syphilis (35), with a significant increase in the degree of inflammatory infiltration in primary lesions from rTp0954/FA-immunized animals when compared with PBS or FA controls (**Figures 7A–F**). Consistent with this observation, rTp0954/FA-immunized rabbits had the lowest levels of treponemal burdens in the primary skin challenge sites and the distal disseminated infection sites, including blood, liver and spleen.

## Discussion

The development of syphilis vaccine was severely hampered by the fact of the fragility of *T. pallidum* outer membrane, its inability to be cultured *in vitro* for a long time, the lack of a system for genetic manipulation to test gene function as well as the scarcity of *T. pallidum* outer membrane proteins (29, 30). Protection of several *T. pallidum* adhesins, including Tp0155 (36), Tp0483 (36), Tp0751 (15, 37), Tp0435 (38) and Tp0136 (28), has been evaluated previously, but only Tp0136 (28) and Tp0751 (15) immunization induced partial protection in NZW rabbits (although Tp0751 protection is controversial) (37). Possible reasons for the failure of other adhesins to induce protection are that (1) the recombinant molecules used for immunization are likely not to maintain native conformation (29, 30) (2), they are not authentic surface-exposed proteins due to difficulties in identifying outer membrane proteins, or (3) they are expressed at low levels or transiently during *T. pallidum* infection (36–38). Therefore, more *T. pallidum* adhesins need to be evaluated for their immune protection. Previous studies suggest that Tp0954 could be a conserved, immunogenic surface-exposed lipoprotein adhesin readily expressed by *T. pallidum* (11, 31, 32), which is conducive to its development as a syphilis vaccine candidate against infection caused by various strains of *T. pallidum*.

It is generally accepted that complete elimination of *T. pallidum* infections relies on both adaptive cellular immunity as well as humor immunity (14, 29, 30). In the early infection stage, the Th1-type cytokine response such as IFN-γ activates resident macrophages, which is considered to be the critical mechanism of clearing *T. pallidum* from primary infection sites (39). The subsequent specific antibodies to *T. pallidum* facilitate opsonophagocytosis of treponemes by macrophages (40–43). In the current study, the histological analysis indicated an obvious increase in the infiltration of lymphocytes, macrophages and differentiated B cells (plasma cells), characteristic of syphilis (35), in primary infection sites of Tp0954-immunized animals. We evaluated neither the activation status nor phagocytic activity of the local cells in primary lesion sites. However, in Tp0954-immunized rabbits, a much higher level of Th1 cytokine IFN-γ from splenocytes and proliferative response specific to rTp0954, as well as increased inflammatory infiltrations of lymphocytes and macrophages, indicate that an effective cellular immune response to *T. pallidum* was developed at the local infection site, which could lead to phagocytosis of spirochetes by resident macrophages activated by IFN-γ produced predominantly by effector CD4^+^ Th lymphocytes. In addition, in Tp0954-immunized animals, high titers of Tp0954-specific serum antibodies, as well as increased infiltration of plasmocytes suggestive of local Tp0954-specific antibody production in primary lesions, could provide protection against *T. pallidum* by 2 proposed mechanisms. One is that the specific antibodies against *T. pallidum* facilitate maintaining treponemes at the initial infection sites by preventing treponemal entry into the vasculature through blocking Tp0954-mediated treponemal attachment to ECM and endothelium. The other mechanism could be that the antibodies promoted opsonophagocytosis of treponemes by macrophages since Tp0954 proved to be a surface-exposed outer membrane protein (11). Collectively, it is likely that both cellular immunity as well as humor immunity contribute to the elimination of *T. pallidum*.

An effective syphilis vaccine requires prevention of chancre development, treponemal transmission and persistence, as well as reinfection so as to eliminate syphilis symptoms in infected individuals and stop the spread of syphilis between people (14). In this study, Tp0954-immunized rabbits showed smaller lesion diameter, shorter hardening time, and lower ulcer incidence at primary lesion sites than control animals, indicating that Tp0954 immunization attenuated cutaneous lesion development. Consistent with these results, qPCR analysis also displayed significantly lower *T. pallidum* burdens at the primary lesion site, which may be due to the clearance of *T. pallidum* by increased infiltrating cells through the mechanisms described above. Since an important property of *T. pallidum* is vascular dissemination, effective syphilis vaccination also requires inhibiting *T. pallidum* in the vasculature spreading to the site of distal infection. Further qPCR analysis showed that the treponemal load in blood, livers and spleens in the immunized rabbits was significantly lower than that of the control animals, indicating that Tp0954 immunization could also inhibit *T. pallidum* dissemination to distant organ sites. Since PCR results cannot distinguish between dead and alive *T. pallidum*, we further confirmed the existence of viable treponemes by the rabbit infectivity test *via* popliteal lymph node transfer. The naive rabbits receiving PLNs from the *T. pallidum-*challenged animals immunized with Tp0954 developed neither orchitis nor seroconversion post transfer during the observation period of 100 days, and did not have any treponemes present in testes extracts *via* silver stain analysis. Conversely, two PLN recipients from PBS control rabbit seroconverted, developed orchitis and had treponemes present. These results further indicated that Tp0954 immunization could inhibit the dissemination of *T. pallidum*. Although Tp0954 immunization did not induce sterile protection at distal infection sites of the Tp0954-immunized animals, Tp0954 immunization could prevent treponemal transmission at the population level since the sterile protection was able to be achieved in PLN recipients from Tp0954-immunized animals.

It is worth noting that there are several limitations to this study. Firstly, the statistical power is limited by the small number of animals used in the experiments, especially in the rabbit infectivity test, and the outbred nature of the animals used in this study. However, our study may provide parameters to determine an adequate sample size for future investigations. Secondly, the FA system used in this study, although effective in induction of innate immunity and adaptive T cell-mediated cellular immune response as well as B cell -mediated IgG and mucosal SIgA responses (44, 45), cannot be used in humans due to its side effects (46). Therefore, adjuvants for clinical use in humans need to be evaluated in further studies. Thirdly, the histological analysis in this study was conducted only at day 21 post *T. pallidum* challenge, which does not reflect the dynamic changes in cell infiltration occurring in the primary lesion, and future experiments should examine the cell infiltration at more time points. And finally, in future studies, we will evaluate whether Tp0954 immunization offers broad cross-protection against heterologous *T. pallidum* strains, even though the amino acid sequence of Tp0954 is completely identical among all sequenced *T. pallidum* strains.

In conclusion, our results indicate immunization with Tp0954 produces similar protective effects as the adhesins Tp0751 and Tp136, suggesting that Tp0954 is a promising vaccine candidate. However, due to the complex pathogenic mechanism of *T. pallidum*, it is difficult to induce complete protection with a single immunogen. Future studies may consider the development of a multicomponent vaccine containing Tp0954 and other protective antigens such as Tp0751, Tp136 or TprK. More importantly, a previous study has proved that Tp0954 is a placental adhesin which can promote attachment of *T. pallidum* to the human placental cell line (11), and therefore, it is worthy of further study whether Tp0954 immunization can block the mother-to-fetus vertical transmission of *T. pallidum* to prevent congenital syphilis.

## Data availability statement

The original data of this article will be provided by the authors without reservation.

## Ethics statement

The animal study was reviewed and approved by the Ethics Committee of the University of South China.

## Author contributions

The experiment was designed by YH, DC, FZ, HL and TZ, and completed by YH, DC, YF, XH, PQ and HY. YH and TZ wrote the manuscript. Statistical analysis was conducted by YH, LY, XZ and LC. Histopathological analysis was performed by HL and TZ. All authors contributed to this article and approved the submitted version.

## Supplementary material

Supplementary Table 1 Serological analysis of naïve recipient animals following popliteal lymph node transfer. R: reactive WR: weakly reactive

Supplementary Figure 1 Silver staining analysis of *T. pallidum* in testicular exudate from different recipient rabbits. Representative silver staining micrographs (1000× magnification) of the testicular exudate from recipient rabbits transferred with PLNs from PBS-immunized control animals on day 56 post-PLN transfer **(A)**, the black arrow indicates *T. pallidum* **)** and from rTp0954/FA-immunized animals on day 100 post-PLN transfer **(B)**.
