# Supplementary figures and images for: Immunization with Tp0954, an adhesin of Treponema pallidum, provides protective efficacy in the rabbit model of experimental syphilis
Source: Front Immunol. 2023 Mar 9;14:1130593. doi: 10.3389/fimmu.2023.1130593 (PMC10042077; doi:10.3389/fimmu.2023.1130593)

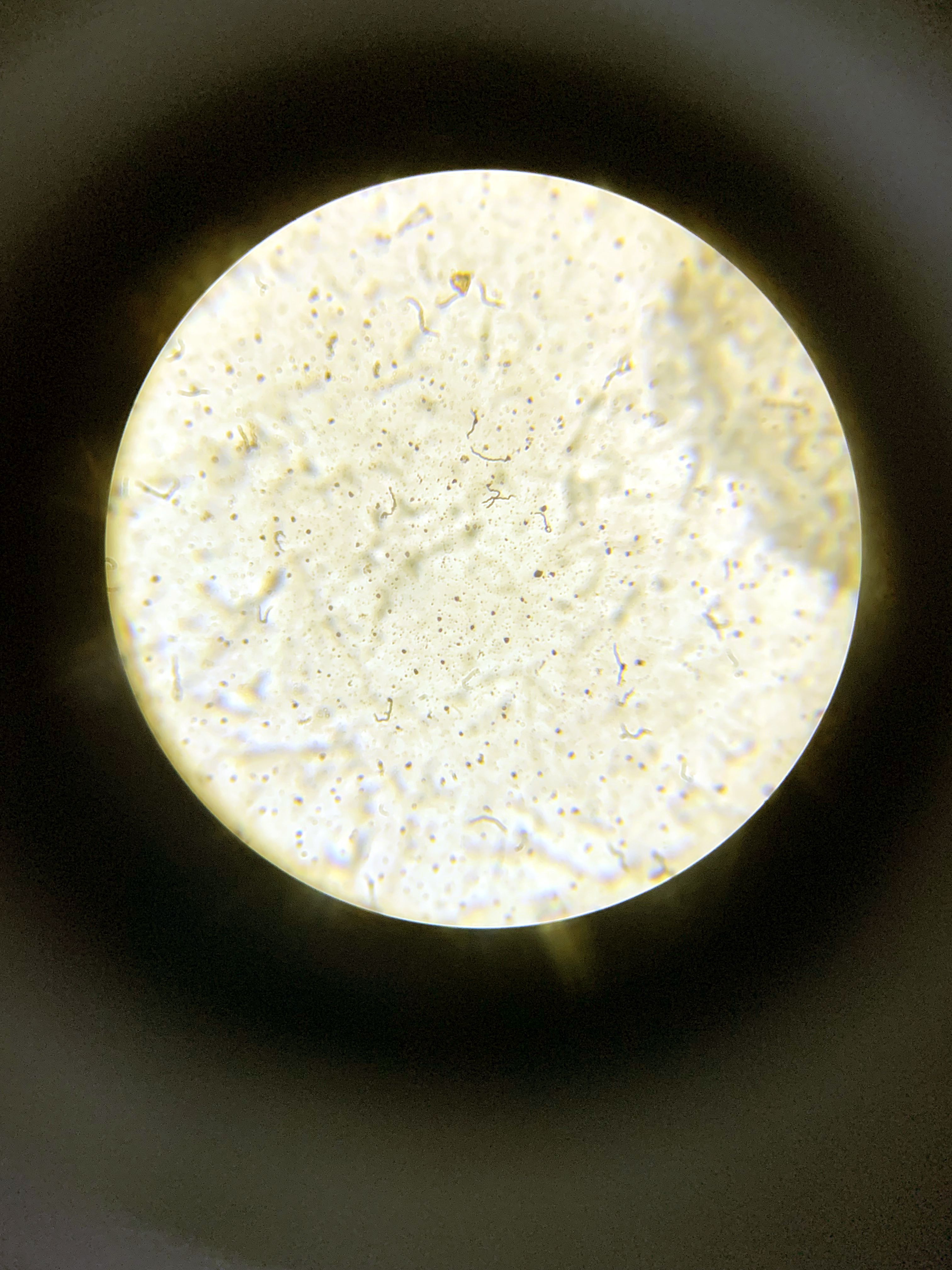

Supplement: Supplementary Table 1 — Serological analysis of naïve recipient animals following popliteal lymph node transfer. R: reactive WR: weakly reactive [file DataSheet_1.zip › 1/Fig S1A.tiff]

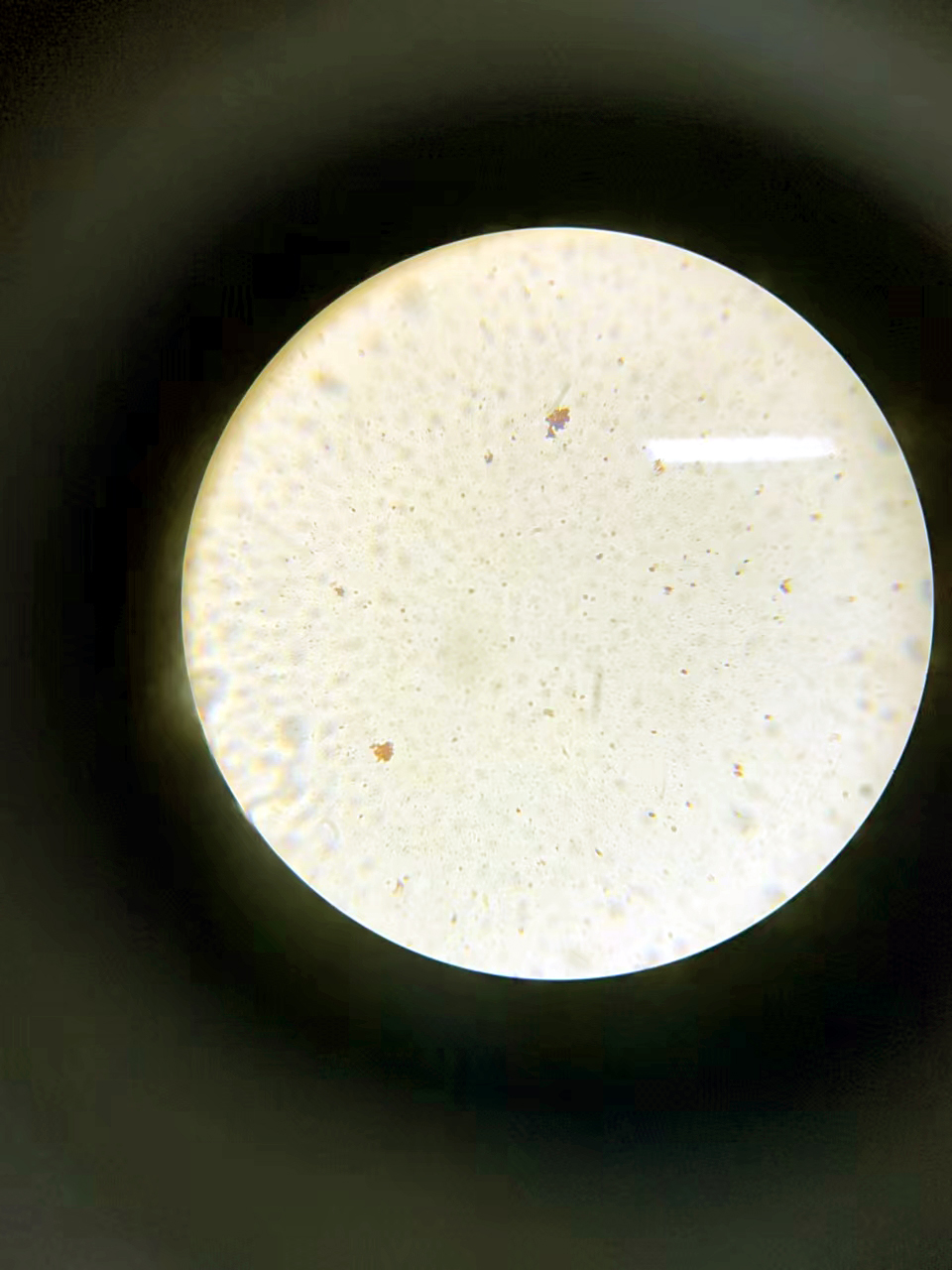

Supplement: Supplementary Table 1 — Serological analysis of naïve recipient animals following popliteal lymph node transfer. R: reactive WR: weakly reactive [file DataSheet_1.zip › 1/Fig S1B.tiff]

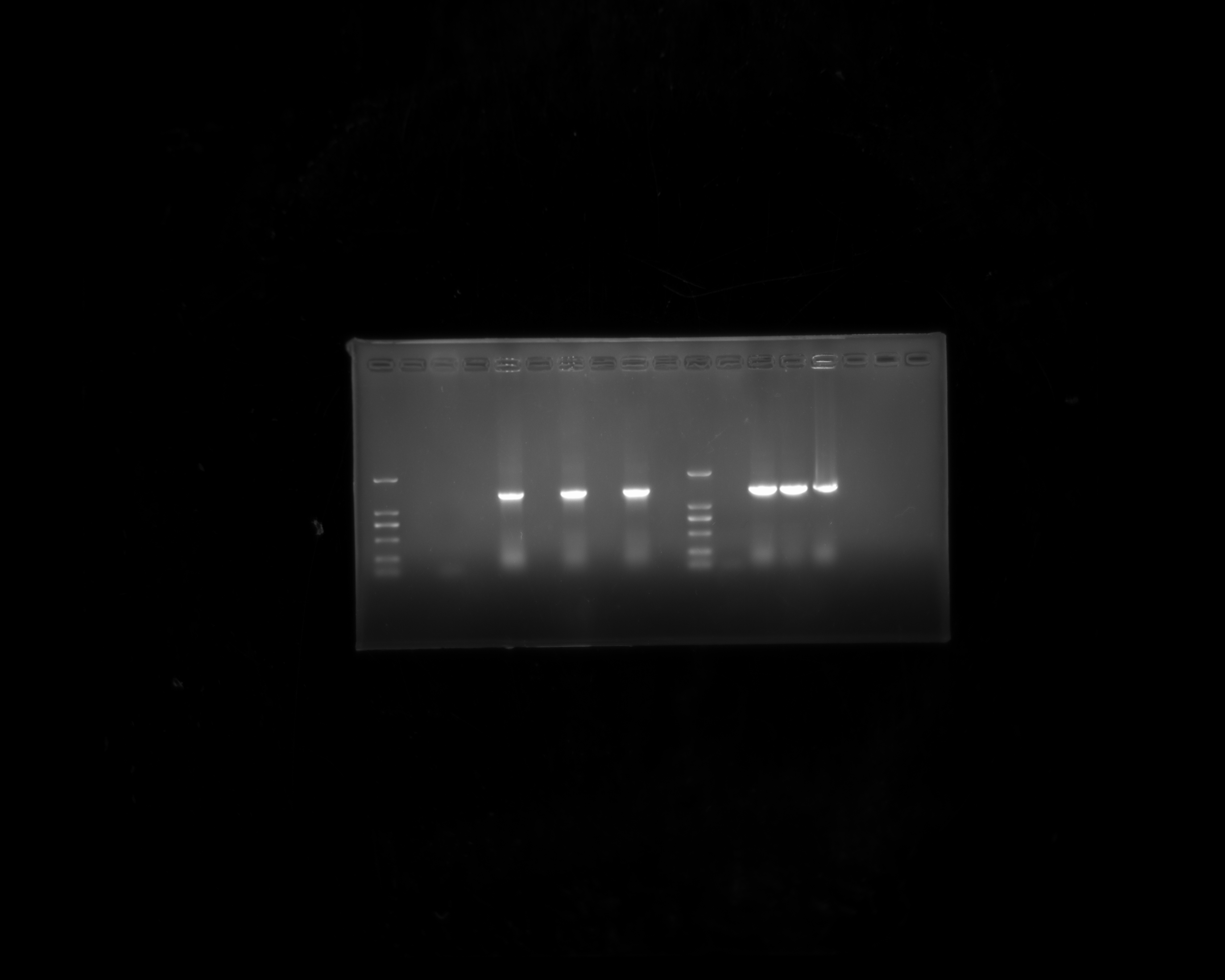

Supplement: Supplementary Table 1 — Serological analysis of naïve recipient animals following popliteal lymph node transfer. R: reactive WR: weakly reactive [file DataSheet_1.zip › 1/Fig1.A.tif]

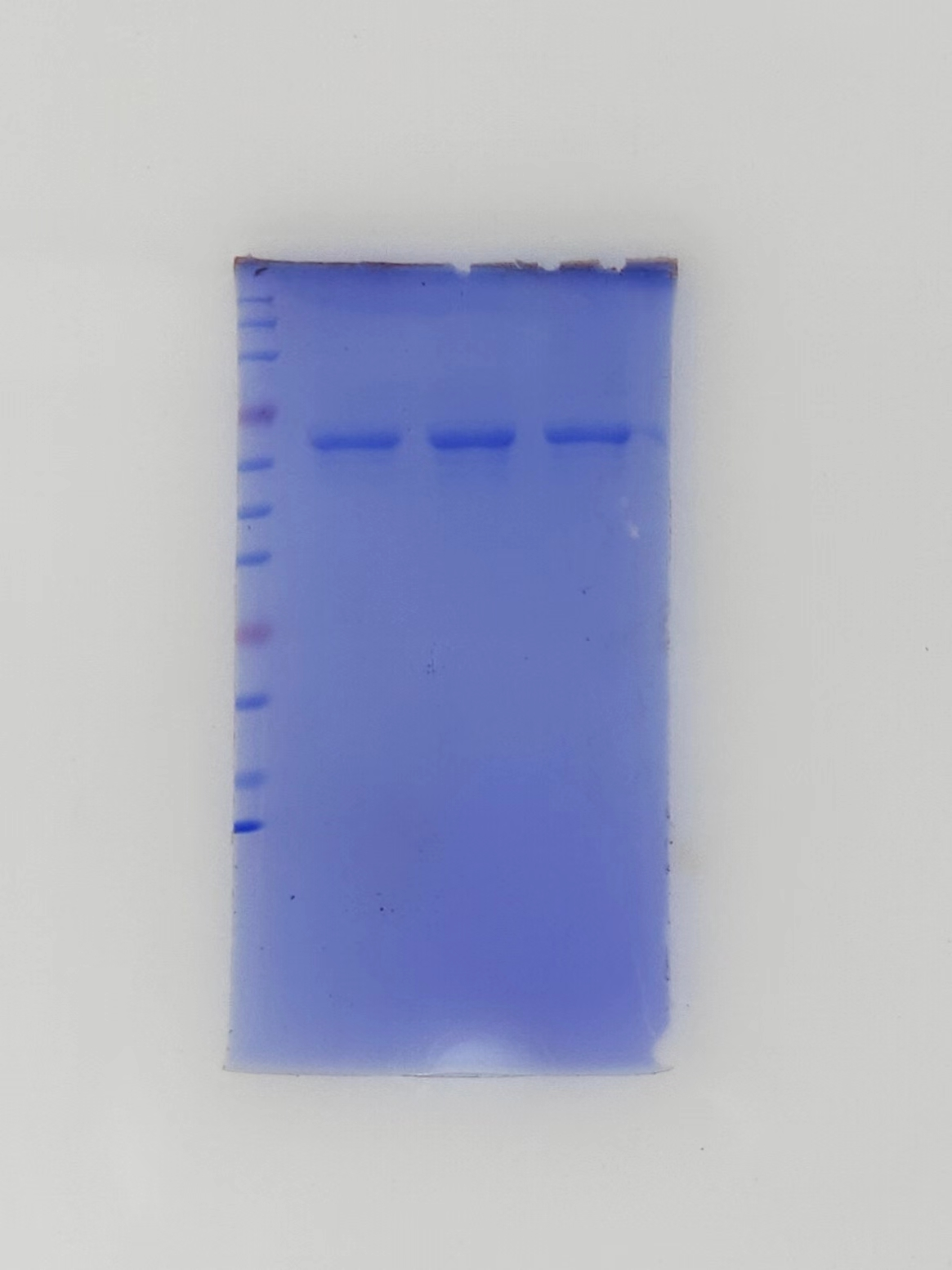

Supplement: Supplementary Table 1 — Serological analysis of naïve recipient animals following popliteal lymph node transfer. R: reactive WR: weakly reactive [file DataSheet_1.zip › 1/Fig1.B.tiff]
